# Comparison of Paracetamol (Apotel®) and Morphine in Reducing Post Pure Head Trauma Headache

**DOI:** 10.5812/aapm.14903

**Published:** 2014-06-21

**Authors:** Samad Shams Vahdati, Hamid Reza Morteza Baghi, Jaffar Ghobadi, Rouzbeh Rajaei Ghafouri, Paria Habibollahi

**Affiliations:** 1 Emergency Department, Medical Faculty, Tabriz University of Medical Sciences, Tabriz, Iran; 2 Drug Information Center and Pharmacy, Faculty of Pharmacy, Tabriz University of Medical Sciences, Tabriz, Iran

**Keywords:** Post Trauma Headache, Paracetamol, Morphine

## Abstract

**Background:**

This randomized, clinical trial evaluates the analgesic and safety of paracetamol and Morphine in management of headache.

**Objectives:**

This study aimed to evaluate the analgesic and safety effects of intravenous single dose of paracetamol, versus morphine in post trauma headache in emergency departments.

**Patients and Methods:**

This study was a single-center, prospective, randomized, double-blind clinical trial conducted on two groups treated with intravenous paracetamol and intravenous morphine. Thirty patients were enrolled in each group. Patients (18-55 years-old adults) complaining from headaches due to pure trauma were included in the study. The inclusion criteria required patients to have headachesof more than 40 mm on a 100 mm visual analogue scale without any pathological findings in their clinical examinations and imaging studies.

**Results:**

Mean duration required to treat the headache was 37.43 and 71.93 minutes in the groups administered paracetamol (group A) and morphine (group B), respectively. After 15 minutes of treatment, this changed to 31.7 ± 18.0 mm (95% CI 8.2 to 25.2) and 48.3 ± 14.1 mm (95% CI 8.2 to 25.2) in groups A and B, respectively. Headache of the patients of group A significantly mitigated in comparison with group B (P < 0.005). Headache of group Apatients was significantly mitigated 30 minutes after treatment (P < 0.005).

**Conclusions:**

Intravenous paracetamol is an effective and safe treatment for patients admitted to the emergency department with headaches caused by head trauma.

## 1. Background

Headache is one of the chief complaints of patients after head traumas and accidents (1). The International Headache Society described PTH (post traumatic headache) in its second edition during 2004, as a headache appearing about 7 days after head trauma (2). To date, there are no studies about the treatment of PTH in adults and children and treatment of headache is only limited to symptom therapy and decreasing headache subjectively. Sometimes, this headache is classified as migraine-like headaches, tension-type headaches, or a combination of both (3). Some medicines including ibuprofen (10 mg/kg) or other nonsteroidal anti-inflammatory drugs (NSAIDs), dexamethasone and morphine are used to treat migraine-like headaches. An efficacious medicine such as tripans will be used if the patient does not respond to these medicines (4, 5). The efficacy and safety of intravenous paracetamol, as an analgesic medicine, has been confirmed (6, 7). According to one study, it was even more effective on tension-type headaches (8). However, it has not been previously evaluated for the management of headache in patients with head traumas.

## 2. Objectives

This study evaluated the analgesic and safety effects of single dose intravenous paracetamol, versus morphine in post trauma headachesof patients admitted to emergency departments.

## 3. Patients and Methods

### 3.1. Study Design

This study was a single-center, prospective, randomized, double-blind clinical trial conducted on two groups treated with intravenous paracetamol and intravenous morphine. The present study was conducted on 60 patients from October 2012 to March 2013. They were randomly divided into two groups; one group was treated with paracetamol (group A) and the other with morphine (group B) and patients were randomly assigned to each group using the Excel software.

### 3.2. Setting

Study participants were from a trauma center of a university hospital (tertiary hospital), to which about 109,500 trauma and non-trauma adult patients are admitted. The study was confirmed by the research deputy of faculty medicine of Tabriz University of Medical Sciences. Written informed consent was obtained from the patients; registration number, IRCT201204127327N2 (Iranian registry of clinical trials; www.irct.ir)

### 3.3. Selection of Participants

Patients (18-55 years-old adults) complaining from headaches due to pure trauma were included in the study. The patients were required to have headaches more than 40 mm on a 100 mm visual analogue scale without any pathological finding in their clinical examinations and imaging studies. Patients with allergy or contraindication to morphine or paracetamol, fever (temperature > 38°C [ 100.4°F], evidence of hemodynamic instability, neurological findings, documented or suspected pregnancy and those that had consumed any analgesic within the last six hours were excluded from the study. Also, patients with documented liver, renal, pulmonary or cardiac disease, transplanted kidney or liver were also excluded. The patients, admitted to the emergency unit during any time of the day and week, were all enrolled in the study. They were evaluated by the attending emergency physician, who considered the inclusion and exclusion criteria of the study. Emphasizing on neurological examinations, all patients underwent brain CT-scans to confirm the lack of pathological findings inside the skull in addition to a history check and physical examination.

### 3.4. Intervention

Participants were blindly and randomly (1:1) divided into two groups to receive either intravenous paracetamol [Apotel 1 gr. (paracetamol 1 gr. made by Unipharm factory, Greece) normal saline (1 g/100 mL/10 minutes)] or morphine (0.1 mg/kg/100 mL/10 minutes NaCl). The randomization table was prepared by one of residents blinded to the study. Medications were prepared by one of the nurses and infused by another nurse who was not aware of the content prepared by the first nurse.

### 3.5. Methods of Measurement

In patients with greater than 40 mm visual analogue pain scale (VAS: this is a tool for evaluation of severity of pain; 0 mm represents no pain and 100 mm indicates the worst pain), headache was measured immediately before receiving the medication and 15 and 30 minutes after administration. The patients were kept uninformed about their headache severity. For better understanding of VAS, the recorder explained the VAS system for the patient and all data was collected by one individual. Medicine side effects mentioned by the patients including nausea, vomiting, agitation, hypotension, vertigo, dizziness, palpitation and cold sweat were recorded. Other probable symptoms were summarized under the title “others”. One week later, a senior resident of the emergency unit followed up the patients by phone, asking whether they had a headache and if so evaluated its rate; because all of the patients understood how VAS works, there was no problem with obtaining the required information by phone.

### 3.6. Outcome Measures

Primary outcome of our study indicates mitigation of headache within the first 15 and 30 minutes. The secondary objective includes recurrence rate of headache after one week.

### 3.7. Primary Data Analysis

The data was analyzed using the SPSS.15.0 statistical system. Mean and standard deviation were determined for the data with normal distribution while middle and interquartile range were specified for data with abnormal distribution. Also, X2 test was run to compare mitigation rate of headache between the two groups after 15 and 30 minutes and one week. Also, X2 test was used to analyze the side effects of the medicines in the two groups.

## 4. Results

Mean age of patients in groups A (paracetamol) and B (morphine) was 37.6 ± 12.5 and 32.9 ± 11.1 years, respectively (P value = 0.46). There were 24 males and 6 females in group B while group A consisted of 18 males and 12 females (P = 0.09).

### 4.1. Main Result

**Figure 1.**
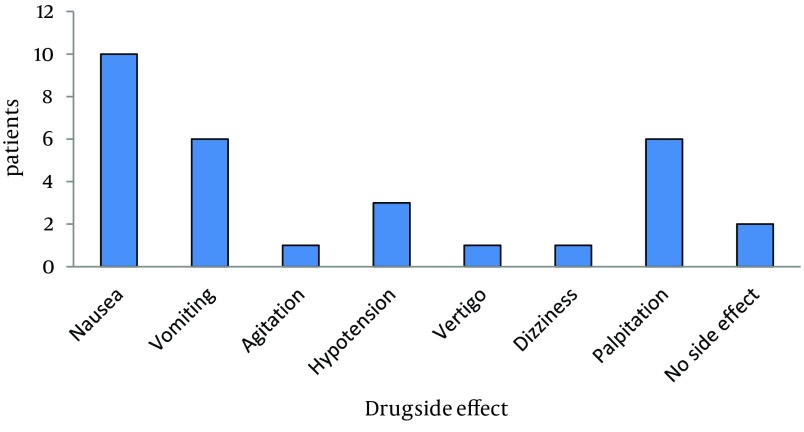
Drug Side Effect Found in the Morphine Group There was no complication or side effect found in group A.

Mean headache severity of patients (by VAS) of group B was 68.7 ± 13.8 mm (95% confidence interval [CI]-8.7 to 4.7), when they referred to the emergency department and 70.7 ± 12.0 mm (95% CI-8.7 to 4.7) for group A. In this regard, there was no meaningful difference between the two groups (P value = 0.552). After 15 minutes of treatment, VAS changed to 31.7 ± 18.0 mm (95% CI 8.2 to 25.2) and 48.3 ± 14.1 mm (95% CI 8.2 to 25.2) in groups A and B, respectively. Headache of the patients of group A significantly mitigated in comparison with group B (P < 0.005). Headache of group A was significantly mitigated 30 minutes after treatment (P < 0.005) (Table 1). Mean duration required to treat headaches was 37.43 and 71.93 minutes in the groups administered paracetamol and morphine, respectively. There was a significant difference between these groups in this regard (P < 0.005). After one week, headache was observed only in one patient of each group. There was no meaningful difference between these two patients (P = 1). The highest rate of side effects was 52.6 (nausea) in group B (Figure 1).

**Table 1.** Numeric Pain Scale of the Two Groups

Time	Numeric Pain Scale ^a^		
	Paracetamol Group	Morphine Group	P Value
**Arrival**	70.7± 12.0	68.7± 13.8	0.552
**After 15 Minutes**	31.7±18.4	48.3±14.2	≤0.0001
**After 30 Minutes**	17.3±15.5	29.0±14.2	0.004
**After one Week**	0.3±1.8	1.3±5.1	0.314

^a^(N = 30).

### 4.2. Limitations

This study was conducted on a limit number of patients with pure head trauma and Glasgow Coma Scale (GCS) of 15. This was regarded as a limitation of the present study. This study could also be conducted on multiple trauma patients. The study followed up the patients only for the first 30 minutes and one week after treatment since immediate pain control is of high importance in emergency management; and also we recommend the new article by greater sample size in some sessions. Nausea and vomiting can be cause of headache and we cannot distinguish it from side effect of Morphine. Additionally, hypotension may return to the trauma. These factors may disturb the study considering side effects of medicines. However, effects of disturbing factors may be alleviated since there are small errors and the patients are randomized.

## 5. Discussion

According to our study, intravenous paracetamol is an efficacious and safe treatment option for patients with post-traumatic headaches in comparison with morphine. This is the first study designed to evaluate effects of intravenous paracetamol in PTH patients in comparison with morphine. Compared with the placebo, paracetamol and its pro-drug have a similar effect in treating physical pains, as stated by Moller et al. Their study reported on greater pain at the injection point of propacetamol than intravenous paracetamol (9).

Murat et al. compared analgesic effects of intravenous paracetamol and propacetamol in 1-12 years-old children undergoing inguinal herniotomy. The study did not use the double-dummy protocol due to ethical considerations because it required injection in both arms. In most double-dummy studies, there was no observed difference considering injection in both arms. In this study, pain at the injection point of propacetamol was more than paracetamol (10). In a prospective orthopedic study on patients undergoing knee and hip-bone surgery, Sinatra et al. found that patients received intravenous propacetamol and paracetamol needed low morphine dosages during the initial 24 hours (11). Also, Van Aken et al. compared the effects of propacetamol, morphineand placebo and concluded that analgesic effects of propacetamol and morphine were better than the placebo. However, there was no difference between propacetamol and morphine considering their analgesic effects (12). Another study compared the effects of tramadol and intravenous paracetamol in patients undergoing heart surgery. According to this study, the patients who received paracetamol experienced less pain within the initial 12, 18 and 24 hours after the operation. Also, they required low dosages of morphine within the initial three days after the operation (13). Bektas et al. introduced intravenous paracetamol as an efficacious and safe medicine to treat renal colic patients at the emergency department (14). Intravenous paracetamol is an effective and safe treatment for patients with headaches caused by head trauma at the emergency department. Intravenous paracetamol represents a good analgesic for emergency settings with low side effects and available as a parenteral agent.

According to this study we need a treatment that is rapid and effective with low side effects for treatment of headache due to trauma and paracetamol is a good choice, as it has all these criteria.
